# Venous thromboembolism after arthroscopic shoulder surgery: a systematic review

**DOI:** 10.1186/s13018-023-03592-0

**Published:** 2023-02-14

**Authors:** Tao Li, Yinghao Li, Linmin Zhang, Long Pang, Xin Tang, Jing Zhu

**Affiliations:** 1 Department of Orthopedics, Orthopedic Research Institute, West China Hospital, Sichuan University, No. 37, Guoxue Road, Wuhou District, Chengdu, 610041 Sichuan Province People’s Republic of China; 2 West China School of Medicine, Sichuan University, Chengdu, People’s Republic of China; 3 Department of Respiratory and Critical Care Medicine, West China Hospital, West China School of Nursing, Sichuan University, No. 37, Guoxue Road, Wuhou District, Chengdu, Sichuan Province People’s Republic of China

**Keywords:** Venous thromboembolism, Arthroscopic shoulder surgery, Systematic review, Prophylaxis

## Abstract

**Purpose:**

To summarize the incidence, risk factors, diagnosis methods, prophylaxis methods, and treatment of venous thromboembolism (VTE) following arthroscopic shoulder surgery.

**Methods:**

Literature on VTE after arthroscopic shoulder surgeries was summarized, and all primary full-text articles reporting at least 1 case of deep vein thrombosis (DVT) or pulmonary embolism (PE) after arthroscopic shoulder surgeries were included. Articles were critically appraised and systematically analyzed to determine the incidence, risk factors, diagnosis, prophylaxis, and management of VTE following arthroscopic shoulder surgeries.

**Results:**

This study included 42 articles in which the incidence of VTE ranges from 0 to 5.71% and the overall incidence was 0.26%. Most VTE events took place between the operation day and the 14th day after the operation (35/51). Possible risk factors included advanced age (> 70 years), obesity (BMI ≥ 30 kg/m^2^), diabetes mellitus, thrombophilia, history of VTE, prolonged operation time, hormone use, and immobilization after surgery. The most common prophylaxis method was mechanical prophylaxis (13/15). No statistical difference was detected when chemoprophylaxis was applied. The management included heparinization followed by oral warfarin, warfarin alone and rivaroxaban, a direct oral anticoagulant.

**Conclusion:**

Based on the included studies, the incidence rate of VTE after arthroscopic shoulder surgeries is relatively low. The risk factors for VTE are still unclear. CT/CTA and ultrasound were the mainstream diagnosis methods for PE and DVT, respectively. Current evidence shows that chemical prophylaxis did not deliver significant benefits, since none of the existing studies reported statistically different results. High-quality studies focusing on the prophylaxis and management of VTE population undergoing arthroscopic shoulder surgeries should be done in the future.

## Introduction

Frequent shoulder dysfunction is the third cause of musculoskeletal consultations in primary health care [1]. The majority of shoulder dysfunction is caused by trauma and degenerative diseases such as arthritis, rotator cuff (RC) injuries and shoulder instabilities [2–5]. For most of those who need surgical therapy, arthroscopic surgery can be a good option since it allows for less trauma to the deltoid, less risk of axillary nerve palsy, less immediate postoperative pain, decreased operation time and better cosmetic results [6, 7]. Besides, recent studies of multi-institutional outcome databases reported low overall rates of complications (1.0–1.6%) and low infection rates after arthroscopic shoulder surgery [8, 9].

With these advantages, the use of the arthroscopic shoulder surgery has expanded greatly [10, 11]. However, despite the application of arthroscopy, venous thromboembolism (VTE), including deep vein thrombosis (DVT) and pulmonary embolism (PE), is still known to be a serious, sometimes even life-threatening, complications following elective upper extremity surgeries [12]. Approximately 20–50% of VTE patients suffered post-thrombotic syndrome, which is detrimental to their quality of life due to chronic hyperpigmentation, edema, pruritus, pain, and ulceration [13].

To reduce and even eliminate the occurrence of VTE, identifying the risk factors is very crucial [14]. Some studies pointed out that comorbidities associated with greater VTE risk include advanced age (≥ 70 years), VTE history, BMI > 40, diabetes mellitus (insulin-dependent and non-insulin-dependent), chronic lung disease and Charlson Comorbidity Index (CCI) ≥ 1 [15–22]. However, little information regarding the risk factors and implementation of preventive measures for VTE after arthroscopic shoulder surgeries have been reported. Therefore, the decision to provide prophylaxis to this population of patients remains subject to the surgeons’ discretion and personal experience. Following an extensive literature search, this systematic review aims to summarize the incidence, risk factors, diagnosis methods, preventive measures and management of VTE after arthroscopic shoulder surgeries and detect the efficacy of chemoprophylaxis in these cases. The hypothesis was that the risk factors for VTE after arthroscopic shoulder surgeries were similar to those mentioned above and the chemoprophylaxis was unnecessary in these cases.

## Methods

### Search strategy

This systematic review was performed following the Preferred Reporting Items for Systematic Reviews and Meta-Analyses (PRISMA) guidelines. The PubMed, Embase, Cochrane Library and Web of Science databases were queried using the following strategy: (“Arthroscopy”[MeSH] OR “shoulder arthroscopy” OR “shoulder”[MeSH]) AND (“Venous Thromboembolism” OR “VTE” OR “deep vein thrombosis” OR “DVT” OR “Upper Extremity Deep Vein Thrombosis” OR “Pulmonary Embolism” OR “vein embolism” OR “pulmonary thromboembolisms” OR “PE” OR “Venous Thromboembolism”[MeSH] OR “Venous Thrombosis”[MeSH] OR “Upper Extremity Deep Vein Thrombosis”[MeSH] OR “Pulmonary Embolism”[MeSH]). The keywords were restricted to the title or abstract. The search was conducted on September 5, 2021.

### Study selection and quality assessment

Two reviewers screened and assessed the studies independently. Two senior authors (one shoulder surgeon and one pulmonary disease expert) reviewed discrepancies and made the final decision. The inclusion criteria for the studies were as follows: (1) with postoperative complications after arthroscopic shoulder surgeries reported; (2) published in a peer-reviewed journal; (3) published in English and (4) full text available. The exclusion criteria for the studies were as follows: (1) basic science studies; (2) only abstracts, review articles or editorial comments; (3) animal or cadaveric studies; and (4) incomplete data. Based on these inclusion and exclusion criteria, the title and abstract of each of the papers were screened first, and the full texts of potentially relevant studies were subsequently reviewed. For those studies with data from the same public databases, the reviewers reached a consensus that only the more recent studies would be included lest some patients be counted repeatedly.

Based on the results of previous literature search, no randomized study on this topic was retrieved. The quality of case series, case control studies and cohort studies was evaluated using the methodological index for non-randomized studies (MINORS), which was designed to assess the quality of both comparative and non-comparative studies. MINORS contains 8 items for non-comparative studies and 12 for comparative studies. Each item is scored 0 (not reported), 1 (reported but inadequate) or 2 (reported and adequate) [23]. As for case reports, Joanna Briggs Institute (JBI) Critical Appraisal checklist was adopted. JBI Critical Appraisal checklist for case reports contains 8 items. Each item has 4 grades, which are *yes*, *no*, *unclear* and *not applicable*. If more than one of the items was rated as *no*, then the study would be excluded. The two independent reviewers appraised the quality of included studies, and any disagreements were resolved by the senior researchers.

### Data extraction

Data from included studies were extracted into the excel sheets by two reviewers. VTE events included DVT (total, proximal, and distal), PE or both, up to 6 weeks post-discharge. The proximal DVT included thrombus in popliteal or common femoral vein and the distal ones included those in the distal part of the popliteal vein (tibial and peroneal veins). PE was defined as having thrombus in the segmental or larger arteries of lungs. The extracted data included blind methods, surgical procedures, number of patients, number of VTE complications, diagnostic methods of VTE, prophylaxis methods of VTE, VTE management and efficacy, mortality, follow-up time and other VTE-related information.

## Results

### Study selection and quality assessment

**Fig. 1:**
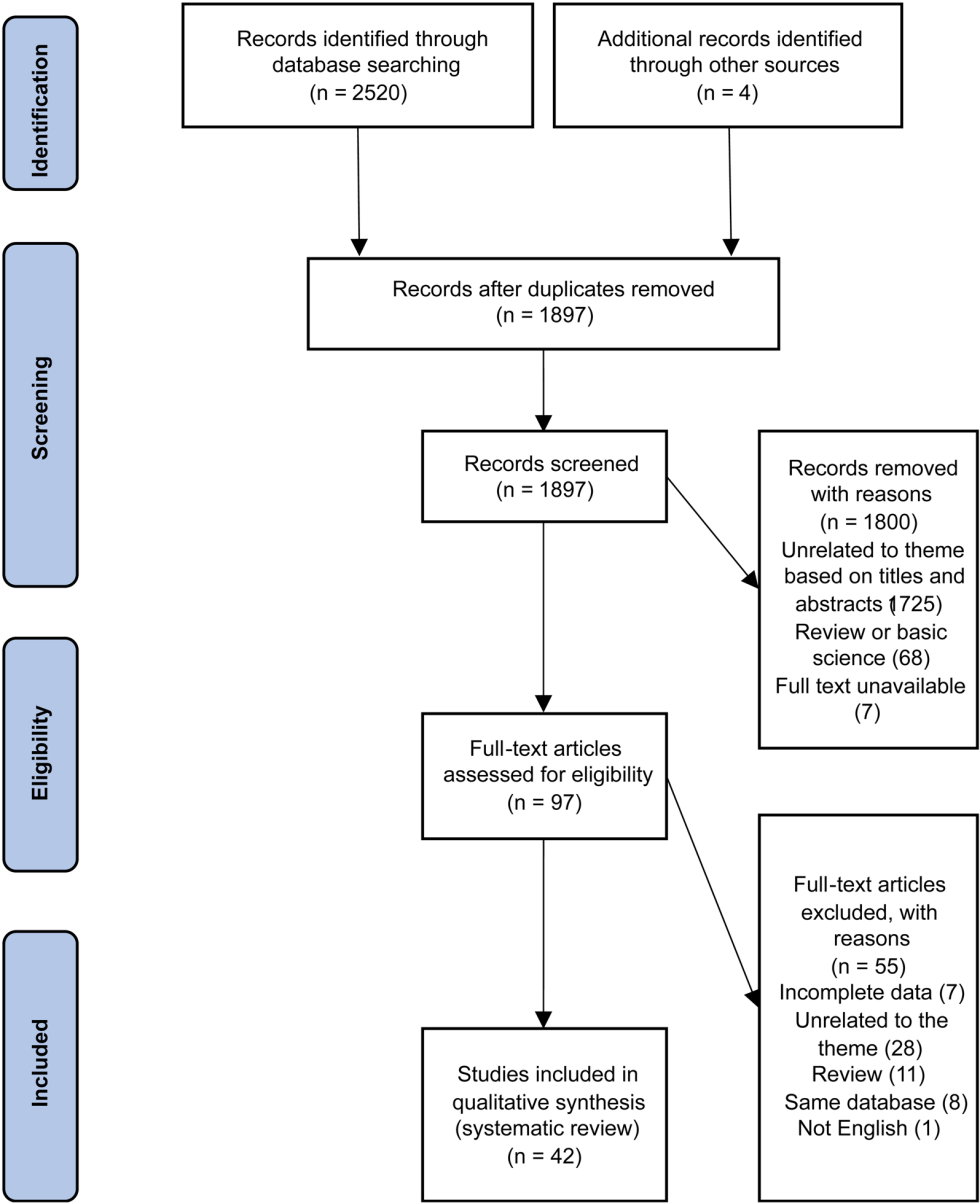
Preferred Reporting for Systematic Reviews and Meta-Analyses study selection flow diagram

The results of our literature search are shown in Fig. 1. The search resulted in 2524 potentially relevant titles, including 627 duplicate articles. After the screening of the abstracts for relevance, we analyzed the remaining 97 full-text articles based on the predetermined inclusion criteria. A total of 42 articles met the inclusion criteria and were included in this systematic review. Among the 42 studies, there were 2 prospective study [24, 25], 17 retrospective studies [26–42] and 23 case reports [43–65].

Five of the retrospective studies specifically focused on the complications of after shoulder arthroscopy [27, 35, 39, 41, 42]. In the remaining retrospective articles, VTE was reported as complications but was not the focus of the study [26, 33, 34, 36–38, 40]. No randomized control trials were identified. The quality assessment of the case reports showed that there was no more than one item in JBI checklist graded as no. Table 1 shows the summary of all the studies included. The MINORS appraisal scores as well as the features of the other studies are listed in Table 2. The risk of bias across the studies is at a moderate level, and the main concern is that most studies were retrospective.

**Table 1:** Summary of included studies

Author	Year	Indications	Procedures	Number of patients	Number of VTE	VTE		Mortality*
						DVT	PE	
Burkhart	1990	Fraying of the anterior glenoid labrum	Synovial resection	1	1	1	0	0
Polzhofer	2003	Synovitis	SAD	1	1	0	1	0
Cortés	2007	RCT	RCR and acromioplasty	1	1	0	1	0
Creighton	2007	SLAP lesion	Labrum repair	1	1	1	1	0
Brislin	2007	RCT	RCR	263	1	1	0	0
Hoxie	2008	RCT	RCR	309	2	0	2	0
Bongiovanni	2009	SLAP lesion and RCT	Labrum repair and RCR	3	3	3	0	0
Hariri	2009	Posterior instability	Posterior capsuloplasty	1	1	0	1	0
Molin	2010	Subchondral cysts and RCT and biceps tendon lesion	RCR and tenotomy on long head	1	1	0	1	0
Garofalo	2010	Recurrent glenohumeral instability and RCT	RCR and others	2	2	2	1	0
Kim	2010	RCT	RCR	1	1	1	1	1
Flanigan	2010	RCT and others	SAD and debridement	13	0	0	0	0
Randelli	2010	All kinds	All kinds	9385	6	5	1	NM
Delos	2011	RCT and synovial debris	Synovectomy and SAD	1	1	1	0	0
Laubscher	2011	RCT	Tenotomy of biceps tendon	1	1	1	1	0
Jameson	2011	All kinds	All kinds	65,302	7	3	5	NM
Duralde	2011	RCT	RCR	53	1	0	1	0
Kuremsky	2011	RCT and labral fraying	RCR and labrum repair	1908	6	4	5	0
Edgar	2012	RCT and SLAP lesion	RCR and labrum repair and SAD	3	3	0	3	0
Yamamoto	2013	RCT	RCR	1	1	0	1	0
Martin	2013	All kinds	All kinds	9410	14	8	6	NM
Durant	2014	RCT	RCR and SAD	5	5	0	5	2
Durant	2014	Labral tear and labral cyst	Labral repair and excision of cyst	1	1	1	0	0
Goldhaber	2014	RCT and SLAP lesion	RCR	1	1	0	1	0
Manaqibwala	2014	RCT	SAD and debridement of RC and RCT	5	5	5	0	0
Ji Yong Gwark	2014	RCT	RCR	1	1	0	1	0
Schick	2014	NM	NM	66	22	15	8	0
Takahashi	2014	RCT	RCR	175	10	10	0	NM
Imberti	2015	All kinds	All kinds	982	3	2	1	NM
Owens	2015	RCT	RCR	2918	6	4	2	NM
Matthews	2017	RCT and Bankart lesion	RCR and Bankart repair	1	1	0	1	0
Yagnatovsky	2017	SLAP lesion	SAD and labrum repair	1	1	0	1	0
Watanabe	2017	Anteroinferior labrum injury	Bankart repair	1	1	1	1	0
Mirzayan	2018	Shoulder pain	Biceps tenodesis mainly	192	2	2	0	NM
Rubenstein	2018	All kinds	All kinds	26,509	66	31	35	NM
Yeung	2019	RCT	RCR	1725	2	2	0	NM
Sager	2019	RCT	RCR	31,615	94	39	66	NM
Stone	2019	All kinds	All kinds	57,727	328	132	196	NM
Alyea	2019	RCT	RCR	914	6	6	0	0
Rangan	2020	Frozen shoulder	Arthroscopic capsular release	203	1	1	0	0
Chauhan	2021	SLAP lesion and RCT	SAD and biceps tenodesis	1	1	1	1	0
Ji	2021	RCT	RCR and SAD and debridement	2	2	0	2	0

*VTE* venous thromboembolism, *RC* rotator cuff, *RCT* rotator cuff tear, *RCR* rotator cuff repair, *SLAP* superior labrum anterior–posterior, *SAD* subacromial decompression, *NM* not mentioned

*Death caused by VTE

**Table 2:** Features of non-case report research

Author	Year	Study design	Data collection	Blind	Baseline	Confounding variables	Follow-up time	Symptoms	Minors score*
*Studies without comparison group*									
Brislin	2007	Case series	Retrospective	No			90 d	Yes	10
Hoxie	2008	Case series	Retrospective	No			42 d	Yes	10
Flanigan	2010	Case series	Retrospective	No			90 d	NA	10
Kuremsky	2011	Case series	Retrospective	No			NM	Yes	10
Duralde	2012	Case series	Retrospective	No			24 m	Yes	10
Martin	2013	Case series	Retrospective	No			30 d	Yes	10
Imberti	2015	Case series	Retrospective	No			90 d	Yes	10
Sager	2019	Case series	Retrospective	No			30 d	Yes	10
*Studies with a comparison group*									
Randelli	2010	Cohort study	Retrospective	No	NM	Not adjusted	NM	Yes	15
Jameson	2011	Case control	Retrospective	No	NM	Not adjusted	90 d	Yes	15
Takahashi	2014	Case control	Prospective	No	NM	Adjusted	90 d	No	18
Schick	2014	Case control	Retrospective	No	Equivalent	Adjusted	NM	Yes	17
Owens	2015	Cohort study	Retrospective	No	Not equivalent	Not adjusted	30 d	Yes	16
Rubenstein	2019	Cohort study	Retrospective	No	NM	Not adjusted	30 d	Yes	15
Stone	2019	Cohort study	Retrospective	No	Equivalent	Adjusted	90 d	Yes	17
Alyea	2019	Cohort study	Retrospective	No	Not equivalent	Not adjusted	6 m	Yes	16
Yeung	2019	Cohort study	Retrospective	No	Equivalent	Not adjusted	NM	Yes	17
Rangan	2019	RCT	Prospective	Yes	Equivalent	Adjusted	12 m	Yes	22

d, day; m, month; MINORS, the methodological index for non-randomized studies; NM, not mentioned; NA, not applicable; RCT, randomized controlled study

*The full score of MINORS for studies without comparison group is 16 and for studies with comparison group is 24

### Incidence and risk factors

Among the 42 included studies, 19 reported the incidence of VTE, which ranged from 0 to 5.71% [24–42]. The overall incidence of VTE was 0.26% (577/224,636).

VTE risk factors were mentioned in 7 studies [24, 29–31, 35, 38, 41]. Jameson et al. [29] retrospectively collected data from a national database. They drew a conclusion that diabetes, > 70 years old and Charlson score ≥ 1 were the risk factors for VTE after arthroscopic shoulder surgeries by calculating the odds ratio (OR). Using a similar method, Sager et al. [38] reported that duration of surgery (> 80 min), male sex, BMI > 30 kg/m^2^ and ASA III or IV were among the VTE risk factors. While the case–control studies conducted by Schick et al. [41] and Takahashi et al. [24] exclude age, BMI, operation time or smoking habit that were VTE risk factors. Chauhan et al. [64] reported that COVID-19 may be a VTE risk factor as well.

Three patients died from PE after arthroscopic rotator cuff repair in total and all of them were reported in the case reports [52, 56]. These three patients were: (1) a 45-year-old female who had diabetes and a BMI of 27.9 kg/m^2^ and died 1 day after the surgery, (2) a 62-year-old female who underwent an arthroscopic shoulder surgery lasting for 134 min and (3) a 63-year-old male with significant comorbidities who underwent an arthroscopic shoulder surgery lasting for 190 min. These cases were reported by Kim et al. [52] and Durant et al. [56].

### Diagnosis and clinical symptoms

The diagnostic methods for VTE in all the 51 patients were reported in detail [27, 39, 42–56, 58, 62–65]. The most common ones were computed tomography (CT)/computed tomography angiography (CTA)/computed tomography pulmonary angiography (CTPA), which accounted for more than half of the total (22/32) [27, 39, 44, 46, 55, 56, 58, 59, 61–63, 65]. Other methods included pulmonary ventilation or/and perfusion scan (5/32) [45, 47, 50, 51, 54] and angiography (2/32) [49, 52]. All the DVT patients received ultrasound. Given its convenience, cheapness and accuracy, ultrasound is not only used to diagnose symptomatic DVT, but also to find the source of pulmonary artery thrombosis or exclude deep vein thrombosis [39, 45, 47, 52, 54, 59].

Among the 51 patients reported in detail [27, 39, 42–56, 58, 62–65], most VTE events took place within 1–14 postoperative day 14 (35/51). All the patients had symptomatic VTE except the 10 patients reported by Takahashi et al. [24]. Common clinical manifestations of PE included dyspnea (17/32) [27, 39, 42, 45–47, 51, 52, 54, 55, 58, 59, 61–63, 65] and chest/left shoulder/scapular pain (10/32) [27, 46, 50, 55, 59, 62, 65]. The rarer ones included tachycardia (2/32) [61, 63], bloody sputum (1/32) [49] and cardiac arrest (1/32) [44]. Common clinical manifestations of DVT included pain (23/28) [39, 42, 47, 48, 50, 51, 57, 60, 64, 65] and swelling (22/28) [39, 42, 47, 48, 51, 53, 58, 60, 64]. The rarer ones included tenderness (7/28) [43, 53, 60], cold sensation (1/28) [60] and groin discomfort (1/28) [54].

### Prophylaxis and treatments

VTE prophylaxis was employed in 15 of the 51 patients reported in detail and 4 retrospective studies focused on the efficacy of prophylaxis [27, 28, 41, 42, 44, 45, 47, 55, 56, 63, 64]. The prophylaxis was either mechanical or chemical or both. Mechanical prophylaxis was more commonly used (12/15), and the compression devices included thromboembolic deterrent (TED) stockings, foot pumps and intermittent pneumatic compression [27, 44, 47, 55, 56, 58, 63]. Chemical prophylaxis was applied in 4 of the 15 patients [45, 55, 56, 64]. Two of them [55, 56] started taking aspirin before surgery and the other two [45, 64] began to take heparin or enoxaparin for VTE prevention from the operation day.

The efficacy of chemical prophylaxis was studied in three of the retrospective studies, and the same conclusion of no significant improvement was reported [28, 41, 42]. In order to identify the factors that were potentially related to VTE following shoulder arthroscopy, Schick et al. [41] conducted a case–control study with the data acquired from the Association of Clinical Elbow and Shoulder Surgeons (ACESS) group. By means of univariate analysis and multivariate logistic regression model, they reported that neither sequential compression devices nor postoperative anticoagulation use was found to be useful in VTE prevention.

**Fig. 2:**
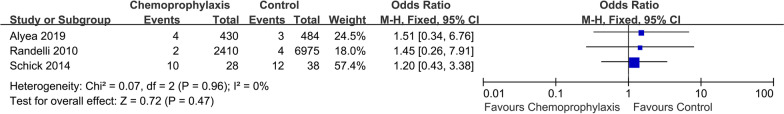
Forest plot for the incidence rate of VTE. *VTE* venous thromboembolism, *CI* confidence interval, *M-H* Mantel–Haenszel

Alyea et al. [42] compared the effectiveness of aspirin and mechanical prophylaxis with mechanical prophylaxis alone in preventing VTE following arthroscopic rotator cuff repair in a retrospective case–control study with 914 patients included. The dosage of aspirin was 81 mg per day and the mechanical prophylaxis included compression boots. Their conclusion was that aspirin application did not reduce the incidence of VTE. In an online survey, Randelli et al. [28] retrieved the data of 9385 surgeries from the members of the Italian Society for Knee Surgery, Arthroscopy, Sports Traumatology, Cartilage, and Orthopaedic Technologies (SIGASCOT). They concluded that using sodium enoxaparin or nadroparin for prophylaxis did not result in a significant reduction in the incidence of VTE. No bleeding events were reported in the research. All the three studies mentioned above showed detailed data, and the results are summarized in Fig. 2.

The treatments of VTE were mentioned in 45 patients though no included studies focused on the efficacy of treatments. The management of VTE typically included heparinization followed by oral warfarin, and this prescription was adopted in 24 patients (53.3%) [27, 42–44, 46, 47, 49, 51, 53–56, 58–61, 63]. However, the dosages of the drugs were not specified and the duration of warfarin application ranged from 6 weeks to 12 months, indicating that such scheme varies from patient to patient. Warfarin alone was adopted in 4 patients [39, 42]. Rivaroxaban, a direct oral anticoagulant (DOAC), was reportedly adopted in 9 patients within the last 7 years [42, 60, 62, 65]. No common complications of anticoagulation like hemorrhage was reported.

## Discussion

To our knowledge, systematic reviews focusing on VTE after arthroscopic shoulder surgeries only are rare, and this study has the largest sample size. Dattani et al. conducted a systematic review to assess the risk factors for and incidence of VTE complications following shoulder and elbow surgeries [66]. However, they discussed not only arthroscopic shoulder surgeries but also open surgeries. Greene et al. focused on thromboembolic complications in arthroscopic surgeries, but the knee instead of the shoulder surgeries was their primary focus [67]. Researchers have illustrated that VTE was rare after shoulder surgery, and it is even fewer after arthroscopic shoulder surgeries than after shoulder arthroplasty [12, 68]. According to this systematic review, there is a large amount of variability in the incidence of VTE after arthroscopic shoulder surgeries reported in the literature, which ranged from 0 to 5.71% [24–41]. The lowest incidence value was shown in a retrospective study, which represented the occurrence of VTE events in anticoagulated patients [33]. The highest incidence value was reported from a prospective cohort study in which 10 asymptomatic VTE events were detected by ultrasound [24]. With 10 large-sample database-dependent studies included, the overall rate of 0.26% is relatively credible [25, 28–32, 35, 37, 38, 41]. This incidence rate demonstrates that the VTE risk for most patients undergoing arthroscopic shoulder surgeries is low. However, surgeons should still be aware of the serious complications in patients after arthroscopic shoulder surgeries due to its potentially fatal risks.

Multiple risk factors are mentioned in the included studies, and the surgery itself elevates the risk of VTE as well. DVT in upper limbs is in majority (60.5%) in this systematic review, while DVT in lower extremities is more common in all patients [69]. A possible theory is that the surgery position may lead to the twisting and stretching of the veins in upper extremities, but it is lacking for validation.

Searching for the risk factors was one of the main focuses of this systematic review. According to previous studies, the risk factors for VTE include advanced age (> 70 years), obesity (BMI ≥ 30 kg/m^2^), diabetes mellitus, thrombophilia, history of VTE, prolonged operation time, hormone use and immobilization after surgery [70]. However, very few studies have attempted to detect the risk factors in patients undergoing arthroscopic shoulder surgeries. Due to the lack of prospective studies, relatively low incidence and conflicting conclusions from different studies, it is difficult to clearly identify and define every certain risk factor. There are several assessment tools to evaluate the VTE risk of patients, but there are also a few articles that dispute them [71]. Establishing a suitable risk assessment tool is one of the goals of future research.

For surgeons, it is obvious that most of the risk factors such as age and existing comorbidities are not controllable, making primary prevention interventions difficult to implement. Based on the existing evidence, the most effective way to reduce the damage of VTE to patients is to evaluate the patients’ risk levels in detail and take corresponding preventive measures for high-risk patients. The education of patients is also very important. The patients should give an explanation of the relevant risks so that they will be able to seek immediate medical attention when they have symptoms of VTE.

In doing this systematic review, we did find that VTE prophylaxis was not provided to most patients. The reasons may be the rarity of the conditions and the fear of bleeding complications. When applied, the prophylaxis was mainly mechanical since they represent the reasonable, safe, and cost-effective option for most patients [68]. Rapp et al. [12] recommended that the efficacious and low-risk mechanical preventions should be used in all patients when feasible. Chemoprophylaxis was used in 5 studies and no bleeding complication was reported, so it seems that hemorrhage is not a concern [42, 45, 55, 56, 64]. However, the efficacy of chemical prophylaxis is doubtable based on this systematic review. A retrospective case–control study conducted by Alyea et al. [42] suggested that the addition of aspirin chemoprophylaxis does not provide protective effect of reducing the incidence of VTE. Schick et al. [41] stated that postoperative anticoagulation use did not show significant influence on VTE development following shoulder arthroscopy. Previous studies and the guidelines in America and Europe did not recommend routine use of chemoprophylaxis in patients undergoing arthroscopic shoulder surgeries unless the patients were assessed to be high risk [12, 70, 72, 73]. Based on the above information, the preferred prevention method we recommend is mechanical prophylaxis, which provides the limb with intermittent pressure, and this can be a routine. The specific method can be selected according to the actual situations of the hospitals and the patients. For high-risk patients, we recommend using DOACs for prophylaxis besides the adoption of mechanical methods [73].

Though the therapeutic regimens varied from study to study, most cases used heparinization followed by oral warfarin, which is different from the recommendations given by the guideline of the American Society of Hematology [74]. In the guideline, DOACs instead of vitamin K antagonists (VKAs) are the first choice for patients with DVT or/and PE if there is not a hemodynamic compromise. This inconsistency can be explained by the time gap between the literature we reviewed, which include studies conducted before September, 2021, and the publishment of the guideline published in 2020. A proof is that cases reported by Ji et al. [65] in 2021 were treated with rivaroxaban or rivaroxaban combined with low molecular weight heparin and the outcomes were good. Therefore, we still recommend following the guideline unless it is proved to be incorrect by further research.

There are several limitations of this study. First, the level of evidence is low since most of the included studies are case reports. However, this is inevitable since there is still a lack of original research with higher level of evidence. Therefore, publication bias as well as other bias was unavoidable. Second, the included studies were inevitably heterogeneous. For these reasons, the conclusion of this systematic review needs to be interpreted with caution. Finally, this study included only the research published in English, so some studies may be missed, whereas this disadvantage did not result in significant bias, given that most high-quality literature around the world is published in English.

## Conclusion

Based on the included studies, the incidence rate of VTE after arthroscopic shoulder surgeries is relatively low. The risk factors for VTE are still unclear. CT/CTA and ultrasound were the mainstream diagnosis methods for PE and DVT, respectively. Current evidence shows that chemical prophylaxis did not deliver significant benefits, since none of the existing studies reported statistically different results. High-quality studies focusing on the prophylaxis and management of VTE population undergoing arthroscopic shoulder surgeries should be done in the future.

## Data Availability

All data generated or analyzed during this study are included in this published article.

## Abbreviations

VTE: Venous thromboembolism
DVT: Deep vein thrombosis
PE: Pulmonary embolism
TED: Thromboembolic deterrent
DOAC: Direct oral anticoagulant
RC: Rotator cuff
CCI: Charlson comorbidity index
PRISMA: Preferred Reporting Items for Systematic Reviews and Meta-Analyses
MINORS: Methodological Index for Non-Randomized Studies
JBI: Joanna Briggs Institute
CT: Computed tomography
CTA: Computed tomography angiography
CTPA: Computed tomography pulmonary angiography
ACESS: Association of Clinical Elbow and Shoulder Surgeons
SIGASCOT: Italian Society for Knee Surgery, Arthroscopy, Sport Traumatology, Cartilage and Orthopaedic Technologies
VKAs: Vitamin K antagonists
